# Initial adaptation and surgical performance of the hinotori™ robotic system among pediatric surgeons with minimal robotic exposure

**DOI:** 10.1007/s00383-025-06212-6

**Published:** 2025-10-02

**Authors:** Serena Iwabuchi, Yuichi Okata, Yasuyuki Kameoka, Harunori Miyauchi, Shohei Yoshimura, Yuki Komyo, Keisuke Kajihara, Yumiko Nakai, Yuko Bitoh

**Affiliations:** Division of Pediatric Surgery, Department of Surgery, Kobe University Graduate School of Medicine, 7-5-2 Kusunoki-Cho, Chuo-Ku, Kobe, 650-0017 Japan

**Keywords:** Hinotori™ surgical robot, Pediatric surgery, Robotic surgery, Simulation training, Surgeon stress, Suturing performance

## Abstract

**Purpose:**

This study aimed to evaluate the ability of the hinotori™ system to mitigate technical and ergonomic challenges in pediatric laparoscopy by comparing usability, suturing precision, and stress responses with that of conventional laparoscopy in a simulated infant abdomen.

**Methods:**

Ten pediatric surgeons with limited robotic experience performed peg transfer and suturing in a 1,400-mL box model simulating a 6-month-old abdomen, using robotic-assisted (Robo) and laparoscopic (Lap) approaches in a crossover design. Performance was assessed using the Fundamentals of Laparoscopic Surgery (FLS) score and the A-Lap Mini platform, which evaluates suturing accuracy across five domains. Physiological stress (heart rate and salivary amylase levels) and subjective fatigue (Chalder Fatigue Scale and visual analog scale) were recorded.

**Results:**

Peg-transfer FLS scores were comparable between Robo and Lap (497.5 vs 531), with progressive improvement across Robo trials. Robotic suturing demonstrated greater air-leak resistance, smaller wound openings, and fewer internal collisions. Stress and fatigue indices did not differ significantly, although trends favored Robo.

**Conclusion:**

Pediatric surgeons with minimal robotic experience achieved higher suturing precision using hinotori™, suggesting intuitive usability and potential safety-related benefits in training. Owing to the small sample size, absence of resident-level validation, and lack of formal performance–stress correlation, confirmation in larger cohorts is warranted.

## Introduction

Minimally invasive surgery has transformed pediatric surgical care by reducing postoperative pain, shortening the length of hospital stays, and minimizing surgical scars. Despite these advantages, performing laparoscopic procedures in neonates and infants remains technically demanding due to the limited working space, restricted instrument mobility, and the lack of depth perception inherent to two-dimensional (2D) visualization systems [1]. These limitations contribute to a steep learning curve and impose substantial mental and physical stress among surgeons.

Robotic-assisted (Robo) surgical systems have been developed to address these challenges by providing enhanced dexterity, tremor suppression, ergonomic improvements, and three-dimensional (3D) visualization [1, 2]. These technological advances have led to the increasing adoption of robotic platforms in adult and pediatric surgeries, with accumulating evidence supporting their feasibility and safety [3–10]. Nevertheless, most commercially available systems, such as the da Vinci® Surgical System, are primarily designed for adult patients and generally require a large operative workspace, thereby limiting their applicability in small pediatric body cavities.

The hinotori™ surgical robot system, developed in Japan in 2019, is equipped with eight-axis robotic arms and a docking-free architecture that enables compact port placement and enhanced flexibility. Its compact footprint and versatile range of motion make it a promising option for pediatric surgery, in which operative space is critically limited.

In a previous simulation study, we demonstrated that the hinotori™ system enables precise suturing within small abdominal cavities (125 mL), simulating the surgical field of neonates [11]. However, that study involved highly experienced engineers certified by the manufacturer rather than clinical surgeons; thus, whether this system can be readily adopted by pediatric surgeons without robotic experience remains unclear. Therefore, this study aimed to assess the usability and adaptability of the hinotori™ surgical robot system among pediatric surgeons with minimal exposure to robotic platforms. By comparing Robo and conventional laparoscopic (Lap) approaches in simulated infant conditions, we aim to evaluate surgical accuracy, performance progression, and physiological and psychological stress responses.

## Methods

### Study design and setting

This prospective crossover study was conducted at the Community Revitalization Center, Kobe University Hospital. The performance, accuracy, and stress responses of pediatric surgeons without formal training and with minimal experience in robotic surgery were compared using the hinotori™ Surgical Robot System (Medicaroid Inc., Kobe, Japan) and conventional laparoscopy under simulated pediatric conditions.

### Surgical platforms and simulated environment

The hinotori™ system comprises a master console and three robotic arms equipped with a 10-mm, 30° videoscope and two 8-mm ports. Laparoscopic tasks were performed using conventional 5-mm instruments and trocars. All procedures were performed using the Endowork Pro II (Kyoto Kagaku Co., Ltd., Kyoto, Japan), a transparent acrylic training box with an internal volume of approximately 1,400 mL. This configuration was selected based on a previous study, which demonstrated that this volume simulates the abdominal cavity of a 6-month-old infant (weighing approximately 8 kg) [11]. Each surgeon performed robotic and laparoscopic tasks, with the order of approaches randomized to minimize potential learning bias.

### Participants

Ten board-certified pediatric surgeons participated in the study. All participants had at least 5 years of surgical experience but no formal robotic training, and most had minimal-to-no prior exposure to surgical robots. The baseline demographics of the participants are presented in Table 1. Written informed consent was obtained from all participants before the study. The protocol was approved by the Institutional Review Board of Kobe University Hospital (IRB No. B250031).

**Table 1:** Baseline demographics of participants

Factor	Median	range
Age	39	[29–60]
Post-graduate year	14	[6–34]
Gender		
Male	9	
Female	1	
Dominant hand		
Right	10	
Left	0	
Average number as operator		
Robot surgery	0	
Laparoscopic surgery		
0–199	4	
> 200	6	

### Surgical tasks

**Fig. 1:**
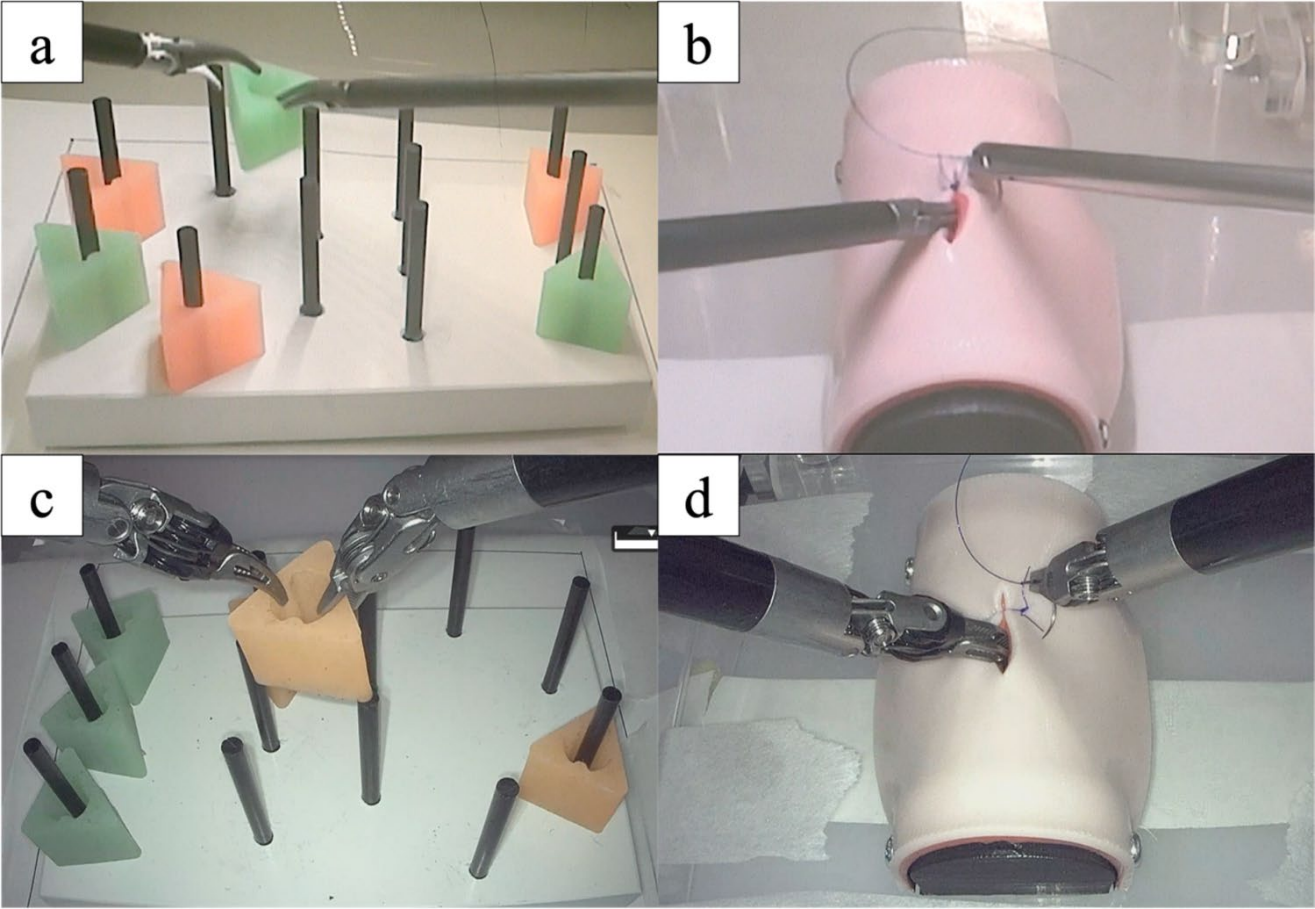
Experimental setup and procedural tasks. **a** Peg-transfer task using conventional laparoscopy. **b** Peg-transfer task using the hinotori™ Surgical Robot System. **c** Intracorporeal suturing task using laparoscopy. **d** Intracorporeal suturing task using the hinotori™ system

Two standardized tasks were performed using both modalities:

Task 1: Peg transfer (Fig. 1a–c):

Adapted from the Fundamentals of Laparoscopic Surgery (FLS) curriculum, this task required participants to transfer six triangular blocks from one side of a 12-peg board to the other and back using Maryland graspers. Each round trip constituted one set, and three sets were completed per trial. A 1-min practice session was allowed before the task.

The FLS score was calculated using the following formula [12–14]:

$$300 - \left( {{\text{seconds}}\,{\text{to}}\,{\text{complete}}} \right) - \left( {10 \times {\text{number}}\,{\text{of}}\,{\text{dropped}}\,{\text{or}}\,{\text{untransferred}}\,{\text{blocks}}} \right).$$

Task 2: Intracorporeal suturing (Fig. 1b–d):

The participants performed three single-knot sutures on a four-layer artificial intestinal model (A-Lap Mini, Kyoto Kagaku Co., Ltd., Japan) using 8-cm-long 5–0 polydioxanone sutures with closed-eye flat needles (Ethicon, Johnson & Johnson, Germany) [15–17]. Each suturing was limited to 5 min, and no prior practice was allowed. All sessions were video-recorded and blindly assessed by an independent pediatric surgeon.

### Outcome measures

#### Performance metrics

For peg transfer, the FLS scores and number of internal instrument collisions were recorded. For the suturing task, accuracy was assessed using the A-Lap Mini system (Kyoto Kagaku, Kyoto, Japan), a computer-assisted device that automatically quantifies suturing performance. The system provides objective scores across five components:

1. Performance time: total time required to complete three stitches
2. Air leak pressure: intraluminal pressure at which leakage occurs following anastomosis.
3. Suture tension: tensile force applied to the suture during knot tying
4. Number of full-thickness sutures: number of stitches penetrating all four layers of the intestinal model
5. Wound opening area: area of dehiscence observed under pressurization, with smaller openings indicating greater suturing precision

The validity and reproducibility of these automated measurements have been demonstrated in previous studies [15, 16].

Each component was rated on a scale of 0–5, yielding a maximum total score of 25. Performance feedback was not provided between tasks.

### Stress and fatigue assessments

Physiological stress was evaluated using heart rate (HR) and salivary amylase activity (SAA), measured pre- and post-task using a fingertip pulse oximeter and portable monitor (Nipro Corp., Osaka, Japan), respectively [18–21]. Subjective fatigue was assessed using the Chalder Fatigue Scale (CFS) and the Visual Analog Scale (VAS) for fatigue [22–24]. Differences (Δ) between pre- and post-task values were calculated for each parameter.

### Statistical analyses

Statistical analyses were performed using JMP Pro version 18 (SAS Institute Inc., Cary, NC, USA) [25]. Median values of paired comparisons (robotic vs. laparoscopic) were analyzed using the Wilcoxon signed-rank test. A p value of < 0.05 was considered statistically significant. Exploratory analyses were carried out to examine the associations between task performance and stress/fatigue indices. Pearson’s correlation coefficients (r) with 95% confidence intervals were calculated, and robustness was assessed using Spearman’s rank correlation (ρ) as SAA values exhibited skewness and potential outliers. To further assess sensitivity, SAA values were winsorized according to the interquartile range (IQR), and the analyses were repeated. As these analyses were exploring, no adjustments for multiple comparisons were applied.

## Results

The 10 participants completed the peg transfer and intracorporeal suturing tasks using Robo and Lap modalities.

### Peg-transfer task

**Fig. 2:**
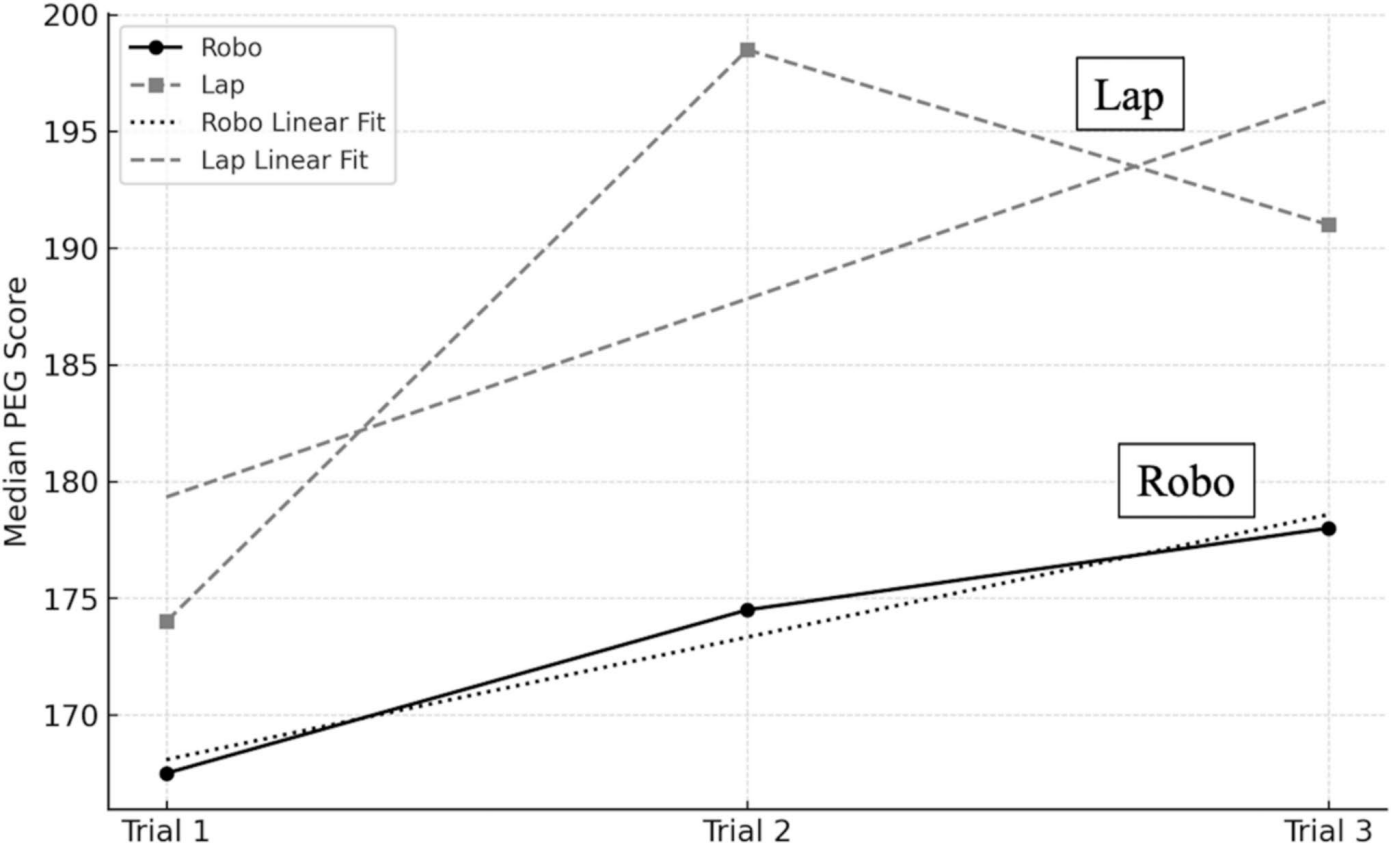
Learning trend of the median peg transfer scores across three trials using the hinotori™ system and laparoscopy. The solid line with circles indicates robotic scores; the dashed line with squares indicates laparoscopic scores. Linear regression lines show progressive improvement, particularly in the robotic group. No statistically significant difference was observed across trials (*p* > 0.05)

FLS scores did not differ significantly between the Robo and Lap groups across the three trials (1st: 167.5 vs 174, *p* = 0.21; 2nd: 174.5 vs 198.5, *p* = 0.15; 3rd: 178 vs 191, *p* = 0.26; total: 519.5 vs 531, *p* = 0.24) (Table 2). Nevertheless, the Robo group demonstrated a trend toward progressive improvement across trials (Fig. 2). The number of internal collisions was significantly lower in the Robo group (1st: 0 vs 2, *p* = 0.01; 2nd: 0 vs 0.5, *p* = 0.38; 3rd: 0 vs 1, *p* = 0.001; and total: 0.5 vs 3.5, *p* = 0.002) compared with the Lap group.

**Table 2:** Results of the skill evaluation of Laparoscopy and Robot: Peg transfer

Variable				Lap		Robo		*p* value
Peg-transfer task				Median	Range	Median	Range	
FLS score			1st	174	[69–217]	167.5	[64–196]	0.21
			2nd	198.5	[125–229]	174.5	[0–206]	0.15
			3rd	191	[83–235]	178	[61–216]	0.26
			Total	531	[313–681]	519.5	[221–618]	0.24
ΔSAA(%)				17	[-67–950]	0	[-97–700]	0.41
ΔHR(/min)				23.5	[3–52]	12	[-8–31]	0.14
ΔVAS(score)				11	[-13–46]	12	[-9–6]	0.25
ΔCFS(score)				0.5	[-7–14]	0	[-2–14]	0.24
Collision (n)	Internal	1st		2	[0–6]	0	[0–2]	0.01*
		2nd		0.5	[0–6]	0	[0–2]	0.38
		3rd		1	[0–2]	0	[0–1]	0.001*
		Total		3.5	[1–7]	0.5	[0–4]	0.002*
	External	Total		0	0	0	0	0

SAA, Salivary Amylase Activity; HR, Heart Rate; VAS, Visual Analog Scale; CFS, Chalder Fatigue Scale

FLS score: A measure from the Fundamentals of Laparoscopic Surgery based on time and dropped pegs,

ΔSAA represents the rate of change, whereas ΔHR, ΔVAS, and ΔCFS denote the difference between pre- and post-task values

^*^p < 0.05

Changes in physiological and subjective stress indicators (ΔSAA, ΔHR, ΔVAS, and ΔCFS) did not differ significantly between modalities. However, the median ΔHR and ΔSAA values were lower in the Robo group compared with the Lap group (ΔHR: 12 vs 23.5, *p* = 0.14; ΔSAA: 0 vs 17, *p* = 0.41), suggesting a potential reduction in physiological stress with Robo.

### Suturing task (A-Lap Mini evaluation)

**Fig. 3:**
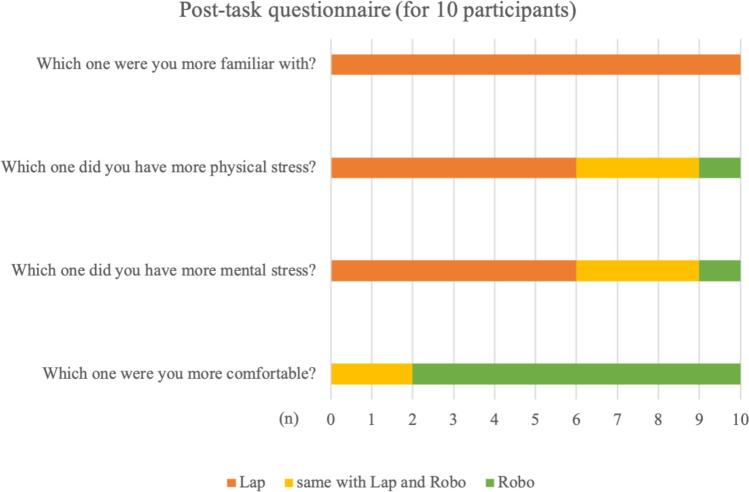
Post-task questionnaire

The Robo group demonstrated higher median total scores compared with the Lap group (14.5 vs. 10.0); however, the difference was not significant (*p* = 0.17) (Table 3 and Fig. 3). Performance metrics favored Robo in leak pressure (2 vs 0, *p* = 0.01) and wound-opening area (5 vs 0, *p* = 0.04), indicating greater suturing precision compared with Lap. Needle handling time was significantly longer in the Robo group compared with the Lap group (264 vs 179, *p* = 0.03); however, the total performance time did not differ significantly between the groups (642.5 vs 559.5, *p* = 0.31).

**Table 3:** Results of the skill evaluation of laparoscopy and robot-assisted surgery: A-Lap Mini

Variable		Lap		Robo		*p* value
A-Lap mini task		Median	Range	Median	Range	
Total score		10	[2 to 18]	14.5	[2 to 19]	0.17
Performance time		2.5	[0 to 4]	2	[0 to 3]	0.21
Volume of air pressure leak		0	[0 to 4]	2	[0 to 5]	0.01*
Suture tension		3	[2 to 5]	3	[0 to 4]	0.28
Number of full-thickness sutures		2	[0 to 5]	1.5	[0 to 5]	0.42
Wound opening area		0	[0 to 5]	5	[0 to 5]	0.04*
ΔSAA (%)		0	[− 86 to 1,267]	0	[− 86 to 325]	0.38
ΔHR (/min)		11.5	[− 9 to 22]	7.5	[− 12 to 43]	0.44
ΔVAS (score)		9	[− 3 to 59]	12.5	[− 4 to 50]	0.40
ΔCFS (score)		1.5	[0 to 14]	0.5	[− 1 to 17]	0.35
Performance time (second)						
Needle handling		179	[109 to 337]	264	[188 to 587]	0.03*
Knot tying		323	[169 to 1,460]	351	[130 to 765]	0.48
Total		559.5	[288 to 1,709	642.5	[352 to 1,063]	0.31
Collision (n)						
Internal	Needle handling	2	[0 to 12]	1	[0 to 3]	0.09
	Knot tying	21	[6 to 61]	11.5	[5 to 20]	0.01*
	Total	22	[8 to 73]	11.5	[5 to 23]	0.01*
External	Total	0	0	0	0	0

SAA, salivary amylase activity, HR: heart rate, VAS: Visual Analog Scale, CFS: Chalder Fatigue Scale

A-Lap Mini task: Each score is graded on a scale of 0–5 points based on task performance

ΔSAA represents the rate of change, whereas ΔHR, ΔVAS, and ΔCFS denote the difference between pre- and post-task values

Performance time is expressed in seconds, whereas collisions are expressed in frequencies

^*^p < 0.05

The number of internal collisions during knot tying was significantly lower in the Robo group compared with the Lap group (11.5 vs 21, *p* = 0.01), with similar results observed for total internal collisions (11.5 vs 22, *p* = 0.01).

No significant differences were observed in stress and fatigue indices (ΔSAA, ΔHR, ΔVAS, and ΔCFS); however, the overall trends were more favorable in the Robo group compared with the Lap group (ΔSAA: 0 vs 0, *p* = 0.38; ΔHR: 7.5 vs 11.5, *p* = 0.44; ΔVAS: 0.85 vs 0.6, *p* = 0.40; and ΔCFS: 0.5 vs 1.5, *p* = 0.35).

Taken together, the robotic platform demonstrated higher air-leak resistance scores (median: 2 vs 0, *p* = 0.016, r = 0.75), superior wound-opening scores (median: 5 vs 0, *p* = 0.046, r = 0.58), and fewer internal collisions during knot tying (median: 11.5 vs 21, *p* = 0.011, r = 0.81) compared with laparoscopy (Tables 2 and 3). These findings are consistent with the collision trends observed in the peg transfer task.

### Post-task questionnaire

Although all participants were more accustomed to laparoscopy, 60% reported greater physical and mental fatigue with Lap, whereas 80% reported greater comfort with Robo (Fig. 3).

### Correlation between performance and stress

Across all 20 trials (Robo + Lap), the FLS score was positively correlated with ΔHR (*r* = 0.676, 95% CI 0.33–0.86, *p* = 0.0011; Spearman’s ρ = 0.769, *p* = 7.3 × 10⁻^5^). Similar correlations were observed within each modality (Robo *r* = 0.732, *p* = 0.016; Lap r = 0.643, *p* = 0.045). No significant correlations were identified between FLS and ΔSAA, ΔVAS, or ΔCFS, nor between A-Lap Mini scores and any stress/fatigue index. Sensitivity analyses addressing SAA outliers did not alter these findings.

## Discussion

To our knowledge, this study is the first to objectively evaluate the accuracy and safety of robotic surgical performance using the hinotori™ Surgical Robot System within a confined space simulating pediatric abdominal conditions and compare these outcomes with those of Lap surgery. The physiological and psychological stress levels in the surgeons were also assessed. The findings demonstrated that pediatric surgeons with minimal prior robotic experience were able to perform suturing more accurately using the hinotori™ system than with laparoscopy. The robotic group achieved superior scores in key parameters, including leak pressure and wound-opening area during intracorporeal suturing, although the completion times were longer. The median total A-Lap Mini scores were higher in the Robo group compared with the Lap group, despite shorter task times in the latter. These results suggest that robotic surgery enables precise performance from the outset, whereas laparoscopy benefits primarily from operator familiarity. A previous study has reported a steep and favorable learning curve with robotic systems [26], indicating that continued training is likely to further improve both efficiency and accuracy. Notably, the number of internal instrument collisions was significantly lower in the Robo group compared with the Lap group during the peg transfer and suturing tasks, highlighting the potential safety advantages of robotic surgery, which is particularly critical in the pediatric setting. These findings are consistent with the reports of previous studies, highlighting the advantages of robotic surgery, including enhanced dexterity, tremor suppression, and 3D visualization. Furthermore, the hinotori™ system may provide distinct advantages in pediatric settings owing to its compact footprint and eight-axis configuration—features that may have contributed to the reduced internal collisions observed in our study. The progressive improvement in peg transfer scores across trials underscores the intuitive operability and trainability of the hinotori™ system. Although participants were experienced with laparoscopy, the steeper learning curve observed in the Robo group suggests that the hinotori™ system can be readily adopted by novice robotic users and is particularly suitable for structured skill acquisition in surgical training.

Stress and fatigue evaluations revealed no significant differences between the groups. However, robotic surgery tended to yield lower ΔHR and ΔSAA values, with a trend toward reduced ΔHR observed during robotic suturing. This pattern, along with consistently lower median ΔSAA, ΔCFS, and ΔVAS values, suggests that robotic surgery imposes less physiological and psychological burden compared with laparoscopy. The ergonomic design of the robotic console and enhanced visualization likely contribute to these benefits. Exploratory correlation analyses indicated that higher FLS performance was positively associated with ΔHR, whereas no adverse correlations were observed for other stress or fatigue measures. This finding is interpreted as reflecting task engagement or arousal rather than detrimental stress, particularly given the improved precision and reduced internal collisions observed during robotic suturing. Nevertheless, this relationship should be interpreted cautiously and validated in larger, multicenter studies.

In the post-task questionnaire, 60% of participants reported greater fatigue with laparoscopy, whereas 80% indicated greater comfort with robotic procedures. These responses support the view that robotic platforms reduce surgeon workload and enhance motivation [27, 28].

The findings of the present study suggest that the hinotori™ system represents a promising platform for clinical pediatric surgery and surgical training. Its ergonomic design, intuitive controls, and ability to reduce internal collisions and operator fatigue make it particularly suitable for integration into pediatric minimally invasive procedures. As robotic systems continue to advance and become more widely accessible, the hinotori™ platform may serve as a valuable alternative to the conventional laparoscopy, particularly in confined pediatric anatomical environments.

This study has some limitations. First, it was conducted as a single-center pilot trial with a small number of board-certified pediatric surgeons, and an a priori sample size calculation was not feasible due to limited availability of the robotic system and eligible participants. Consequently, the statistical power was limited. Hence, the findings should be interpreted with caution. Nevertheless, the observed effect sizes provide useful preliminary estimates, suggesting that approximately 10–20 participants may be required to achieve adequate power in future confirmatory trials. Second, laparoscopy was performed using a 2D imaging system with relatively lower image quality compared with the 3D high-definition view available with hinotori™, which may have influenced performance and stress levels. Third, the use of a simulation box cannot fully replicate the dynamic anatomy and tissue properties of actual pediatric abdominal cavities, particularly in neonates with soft and elastic tissues. Moreover, intraoperative variables, such as organ movement, lighting changes, and emergency scenarios, were not replicated in this model. Finally, this study did not include comparisons with other robotic systems, such as da Vinci®, Versius®, or Senhance™, which should be addressed in future studies.

Future studies should include larger, multicenter cohorts and high-fidelity models, including 3D-printed organs or animal models, to more accurately replicate pediatric anatomy. Comparative evaluations with other robotic systems and long-term studies assessing learning curves and clinical outcomes will be essential for establishing evidence-based guidelines for robotic pediatric surgery.

This study demonstrated that the hinotori™ Surgical Robot System enables the safe and accurate performance of basic and complex surgical tasks under simulated pediatric surgical conditions, even among surgeons with minimal prior robotic experience. Although laparoscopic procedures were completed more rapidly due to user familiarity, the robotic approach yielded higher suturing precision, fewer internal collisions, and lower physiological and psychological stress levels.

The intuitive operability and steep learning curve of the hinotori™ system indicate its suitability for pediatric surgical training and future clinical application. With continued technological refinement and broader validation, the hinotori™ system holds strong potential to enhance safety, precision, and surgeon well-being in pediatric minimally invasive surgery.

## Data Availability

The datasets generated and analyzed during the current study are not publicly available due to the inclusion of personal will be made available by the corresponding author upon reasonable request.
